# Stability of mild cognitive impairment in newly diagnosed Parkinson's disease

**DOI:** 10.1136/jnnp-2016-315099

**Published:** 2017-03-01

**Authors:** Rachael A Lawson, Alison J Yarnall, Gordon W Duncan, David P Breen, Tien K Khoo, Caroline H Williams-Gray, Roger A Barker, David J Burn

**Affiliations:** 1 Institute of Neuroscience, Newcastle University, Newcastle upon Tyne, UK; 2 Centre for Clinical Brain Sciences, University of Edinburgh, Edinburgh, UK; 3 John van Geest Centre for Brain Repair, University of Cambridge, Cambridge, UK; 4 School of Medicine & Menzies Health Institute Queensland, Griffith University, Southport, Queensland, Australia; 5 School of Medicine, University of Wollongong, Wollongong, New South Wales, Australia

**Keywords:** PARKINSON'S DISEASE, DEMENTIA, COGNITION

## Abstract

**Background:**

Mild cognitive impairment (MCI) is common in early Parkinson's disease (PD). We evaluated the stability of PD-MCI over time to determine its clinical utility as a marker of disease.

**Methods:**

212 newly diagnosed participants with PD were recruited into a longitudinal study and reassessed after 18 and 36 months. Participants completed a range of clinical and neuropsychological assessments. PD-MCI was classified using Movement Disorders Society Task Force level I (Montreal Cognitive Assessment <26) and level II (using cut-offs of 1, 1.5 and 2SD) criteria.

**Results:**

After 36 months, 75% of participants returned; 8% of patients had developed a dementia all of which were previously PD-MCI. Applying level I criteria, 70% were cognitively stable, 19% cognitively declined and 11% improved over 36 months. Applying level II criteria (1, 1.5 and 2SD), 25% were cognitively stable, 41% cognitively declined, 15% improved and 19% fluctuated over 36 months. 18% of participants reverted to normal cognition from PD-MCI.

**Discussion:**

Cognitive impairment in PD is complex, with some individuals' function fluctuating over time and some reverting to normal cognition. PD-MCI level I criteria may have greater clinical convenience, but more comprehensive level II criteria with 2SD cut-offs may offer greater diagnostic certainty.

## Introduction

Cognitive impairment in Parkinson's disease (PD) is common, ultimately 80% of patients may develop dementia (PDD).^1^ Mild cognitive impairment in PD (PD-MCI) may be a prodromal stage of PDD. Guidelines to identify PD-MCI have been proposed by the Movement Disorder Society (MDS).^2^ The MDS criteria have yet to be validated, but several recent studies have investigated the number and optimal assessments, and best cut-offs to define PD-MCI.^3^ ^4^

The MDS PD-MCI guidelines specify that in order to meet criteria for this diagnosis, a patient with PD must exhibit gradual cognitive decline (reported by the patient, informant or clinician) that is not severe enough to impair functional independence or activities of daily living.^2^ The patient should not have another primary explanation for their cognitive impairment such as delirium, stroke, major depressive disorder or head trauma. The patient should also not have any other PD-associated conditions that may significantly interfere with cognitive testing (eg, motor impairment, severe anxiety, daytime somnolence or psychosis).

Litvan *et al* 2 classified PD-MCI criteria using either level I criteria (primarily for use in a clinical setting) or more stringent level II criteria (primarily for use in a research setting). Level I criteria require a less comprehensive battery of tests, for example, impairment on a global cognitive test which has been validated in PD such as the Montreal Cognitive Assessment (MoCA), or impairment on at least two tests in a limited battery of neuropsychological tests. Application of level II criteria requires at least two neuropsychological tests across each of five cognitive domains: attention, executive function, visuospatial function, memory and language.^2^ Patients meeting level II criteria should either be impaired in two or more tests in one cognitive domain (single domain PD-MCI) or in at least one test in two or more different domains (multidomain PD-MCI). Impairment is defined as performance of 1–2SD below appropriate norms, significant decline on serial neuropsychological testing, or a decline from premorbid levels. Level II criteria also include optional subtype classification: single domain or multidomain classification as well as type of impairment.

The longitudinal characteristics of PD-MCI are unknown, and whether it is a stable state, likely to decline or even improve over time. A multicentre study investigating the stability of MCI and dementias found that MCI was associated with diagnostic uncertainty.^5^ We hypothesised that PD-MCI may also be associated with prognostic uncertainty, which could be problematic in terms of clinical management, while causing unnecessary distress for patients. This study sought to determine the stability and clinical utility of PD-MCI in newly diagnosed patients with PD over 36 months.

## Methods

This study was approved by the Newcastle and North Tyneside 1 Research Ethics Committee. All participants provided written informed consent.

Recently diagnosed patients with PD were recruited from the community and outpatient clinics in Newcastle and Cambridgeshire, UK as part of the Incidence of Cognitive Impairment in Cohorts with Longitudinal Evaluation in PD (ICICLE-PD) study^6^ ^7^ and were evaluated at 18-month intervals. At each assessment, demographic and clinical data were collected including MDS Unified Parkinson's Disease Rating Scale (MDS-UPDRS) Part III, Geriatric Depression Scale (GDS-15) and levodopa equivalent daily dose (LEDD).^8^

A detailed schedule of neuropsychological tests was performed at each assessment and has been described previously.^6^ Attention was assessed using Power of Attention (PoA) and Digit Vigilance Accuracy from the Cognitive Drug Research Battery.^9^ Executive function was assessed using the One Touch Tower of London (OTS) from the Cambridge Neuropsychological Test Automated Battery (CANTAB),^10^ phonemic fluency and semantic fluency.^11^ Memory was assessed using spatial and pattern recognition memory (SRM and PRM), and paired associate learning (PAL) subsets from the CANTAB. Visuospatial function was measured using modified scoring for copying interlocking pentagons.^12^ Language was measured using the naming and language scores from the MoCA.^13^

We used modified level II criteria to classify PD-MCI as our study design predated the MDS Task Force PD-MCI criteria (described by Yarnall *et al* 6). We applied cut-offs of 1.0 (≥1 but <1.5SD), 1.5 (≥1.5 but <2SD) and 2.0 (≥2SD) SDs below normative values (controls), or an approximation to the normal distribution, to classify PD-MCI. We also applied level I PD-MCI criteria, with cognitive impairment defined as an MoCA score <26.^2^ For both criteria, semistructured interviews were conducted with participants and their carers to determine subjective cognitive symptoms and functional independence. We classified participants according to the MDS criteria: single domain or multidomain PD-MCI and the domains impaired (described by Lawson *et al* 14).

Data were examined for normality, and means were compared using analyses of variance or Kruskal-Wallis tests as appropriate (SPSS V.21.0). The χ^2^ tests were used to compare between-group distributions of proportion. Cochran's Q test compared between-group proportions over time.

## Results

Two hundred and twelve newly diagnosed participants with PD completed baseline assessments; 190 (89.6%) and 158 (74.5%) returned for 18-month and 36-month evaluations, respectively (mean of 3.1±0.2 years). Demographic and clinical characteristics of participants at each time point are presented in table 1. Participants with cognitive impairment tended to be older, had completed fewer years of education, and had greater motor severity and lower global cognition scores (p<0.01 for all).

**Table 1:** Demographic and clinical characteristics of participants delineated by cognitive status at 18-month intervals

	Baseline (n=212)					18 months (n=190)						36 months (n=158)					
	PD-CN (n=73)	PD-MCI 1SD (n=51)	PD-MCI 1.5SD (n=43)	PD-MCI 2SD (n=45)	p Value	PD-CN (n=60)	PD-MCI 1SD (n=43)	PD-MCI 1.5SD (n=26)	PD-MCI 2SD (n=53)	PDD (n=8)	p Value	PD-CN (n=56)	PD-MCI 1SD (n=17)	PD-MCI 1.5SD (n=25)	PD-MCI 2SD (n=44)	PDD (n=16)	p Value
Age (years)	61.2 (10.1)	67.3 (8.4)	69.1 (8.8)	68.7 (9.0)	<0.001*	63.9 (9.3)	67.3 (8.9)	68.7 (9.0)	72.5 (8.2)	74.8 (6.3)	<0.001*	64.2 (8.7)	68.7 (8.9)	69.8 (8.5)	72.3 (8.4)	76.1 (7.4)	<0.001*
Gender (male: n, %)	41 (56.2)	35 (68.6)	28 (65.1)	30 (66.7)	0.359†	32 (53.3)	30 (69.8)	20 (76.9)	35 (66.0)	5 (62.5)	0.233†	1.4 (0.5)	1.4 (0.5)	1.3 (0.5)	1.3 (0.5)	1.3 (0.5)	0.589†
Education (years)	14.5 (3.7)	13.1 (3.3)	11.2 (2.4)	11.3 (3.5)	**<0.001‡**	14.0 (3.3)	12.9 (3.4)	12.7 (3.7)	11.2 (2.9)	12.1 (4.4)	**<0.001‡**	13.9 (3.3)	13.8 (4.2)	12.8 (3.6)	11.9 (2.8)	11.8 (3.8)	**0.001‡**
MDS-UPDRS III	22.7 (9.5)	27.2 (10.4)	32.1 (11.9)	31.2 (14.2)	**<0.001‡**	28.2 (11.0)	30.2 (11.6)	33.2 (9.8)	40.7 (11.8)	48.1 (8.9)	**<0.001‡**	29.0 (12.1)	32.7 (13.6)	38.8 (12.4)	41.1 (14.5)	46.3 (16.3)	**<0.001‡**
Hoehn and Yahr stage	1.7 (0.6)	1.9 (0.6)	2.1 (0.6)	2.1 (0.8)	**0.001‡**	2.0 (0.5)	2.1 (0.4)	2.2 (0.5)	2.4 (0.6)	2.6 (0.7)	**0.005‡**	2.0 (0.4)	2.2 (0.6)	2.2 (0.6)	2.2 (0.6)	2.7 (0.8)	**0.003‡**
LEDD (mg/day)	179.8 (166.4)	149.1 (130.3)	200.1 (180.5)	190.1 (133.8)	0.363‡	415.0 (213.3)	465.1 (270.9)	348.7 (181.8)	434.1 (253.0)	303.1 (123.6)	0.259‡	499.5 (289.1)	562.4 (374.4)	495.6 (202.4)	586.6 (300.5)	472.7 (230.4)	0.834‡
GDS-15	2.3 (1.9)	2.7 (2.9)	2.8 (2.5)	3.8 (3.2)	0.084‡	2.3 (2.5)	2.7 (2.6)	3.0 (2.2)	3.6 (3.1)	3.0 (1.3)	0.102‡	2.4 (2.3)	2.7 (2.3)	3.4 (2.6)	3.5 (2.6)	4.6 (2.6)	**0.013‡**
MoCA§	27.4 (1.8)	26.1 (2.3)	24.5 (3.3)	22.3 (4.0)	**<0.001‡**	28.6 (1.6)	26.6 (2.9)	25.6 (2.7)	24.3 (3.1)	17.4 (3.7)	**<0.001‡**	28.2 (1.9)	27.0 (1.8)	25.8 (2.8)	23.8 (3.2)	19.6 (4.3)	**<0.001‡**
MMSE	29.3 (0.9)	28.8 (0.9)	28.5 (1.3)	28.0 (1.6)	**<0.001‡**	29.4 (0.9)	28.6 (1.3)	28.3 (1.4)	27.6 (1.6)	25.0 (2.2)	**<0.001‡**	29.1 (1.2)	28.8 (1.2)	28.5 (1.3)	27.4 (2.4)	24.8 (3.4)	**<0.001‡**

Figures are mean (SD) unless otherwise stated; significant differences are highlighted in bold.

§At baseline, n=23 did not complete MoCA.

*ANOVA.

†χ^2^ Test.

‡Kruskal-Wallis test.

ANOVA, analysis of variance; GDS-15, Geriatric Depression Scale; LEDD, levodopa equivalent daily dose; MDS-UPDRS III, Movement Disorders Society-Unified Parkinson's Disease Rating Scale Part III; MMSE, Mini-Mental State Examination; MoCA, Montreal Cognitive Assessment; PD-CN, Parkinson's disease with normal cognition; PDD, Parkinson's disease dementia; PD-MCI, mild cognitive impairment in Parkinson's disease.

**Figure 1:**
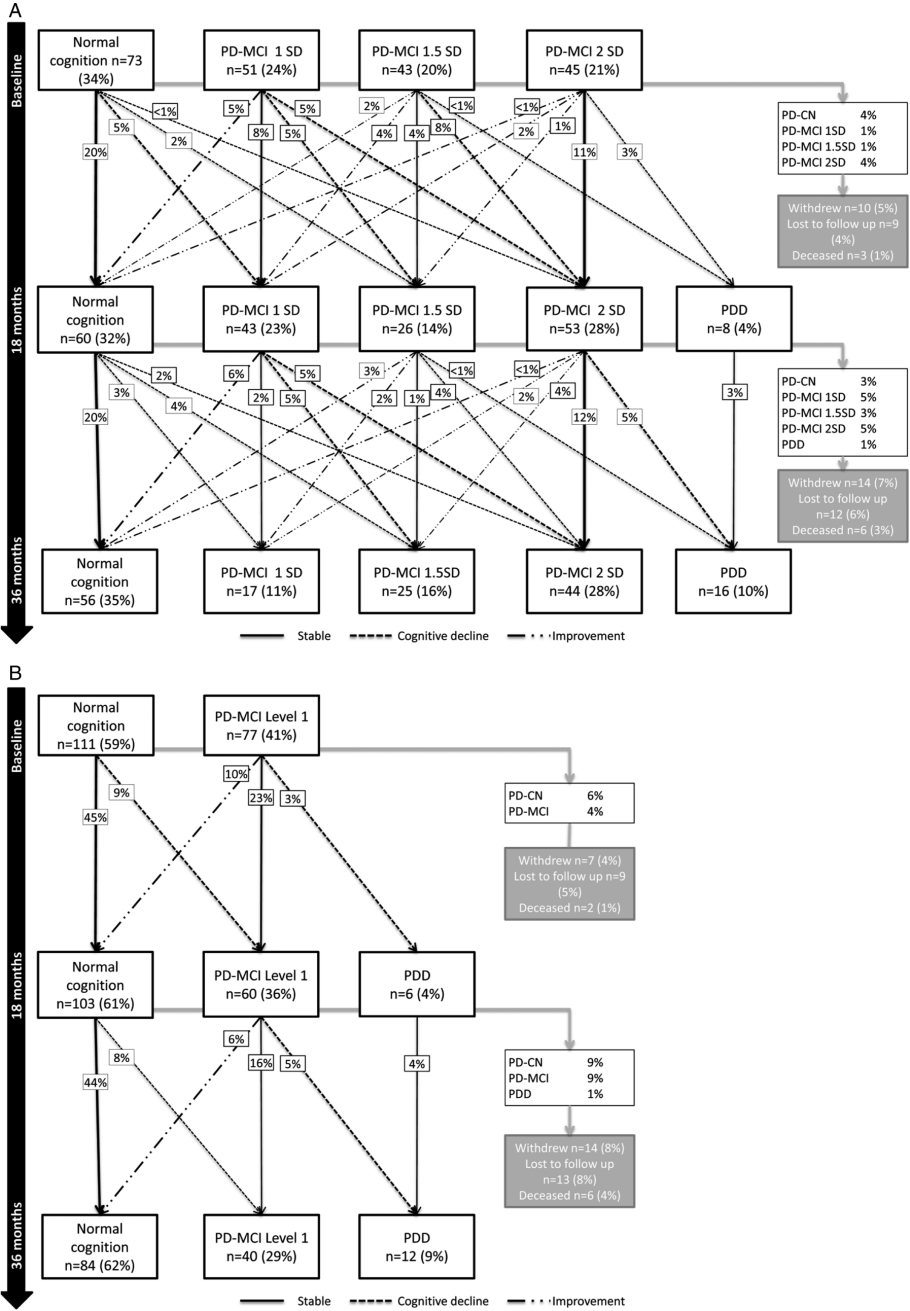
Changes in cognitive classification from baseline to 36 months. (A) Change in cognitive classification using MDS PD-MCI level II criteria to classify PD-MCI using cut-offs of 1, 1.5 and 2SD. (B) Change in cognitive classification using MDS PD-MCI level I criteria to classify PD-MCI using MoCA score. Twenty-three participants did not complete MoCA at baseline and were excluded from this analysis. Percentages relate to the proportion of participants evaluated at that time point: baseline, 18 or 36 months. MDS, Movement Disorders Society; MoCA, Montreal Cognitive Assessment; PD-CN, Parkinson's disease with normal cognition; PD-MCI, mild cognitive impairment in Parkinson's disease; PDD, Parkinson's disease dementia.

Figure 1 shows the changes in cognitive classification between groups at each time point using level II (figure 1A) and level I (figure 1B) PD-MCI criteria. Between baseline and 18 months, 43% had stable cognition, 30% cognitively declined and 15% improved in terms of their PD-MCI classification (1 vs 1.5 vs 2SD) using level II criteria. Between 18 and 36 months, 35% of remaining participants had stable cognition, 29% cognitively declined and 18% improved (figure 1A). Between baseline and 36 months, 27% (n=58) were cognitively stable, 33% (n=71) cognitively declined and 14% (n=29) improved; 8% (n=18) developed PDD.

As shown in figure 1A, the majority of cognitively stable participants were classified as normal cognition (PD-CN), with 20% of participants classified as stable PD-CN at 18 and 36 months. PD-MCI 2SD was the next most stable group, with 11% and 12% being consistently classified as PD-MCI 2SD at 18 and 36 months, respectively. Nineteen per cent of participants fluctuated over time, with the majority of participants fluctuating between PD-MCI 1SD, 1.5SD and PD-CN.

Some participants reverted to PD-CN at 18 (8%) and 36 months (10%) having previously been classified as PD-MCI (cumulatively 18%), although this is <1% at each time point when applying PC-MCI 2SD criteria. Reverters were significantly younger than non-reverters (61.3±9.6 vs 68.2±9.2 years, respectively, p<0.01) and at baseline had better neuropsychological scores for phonemic and semantic fluency, OTS, PoA, PRM and PAL (p<0.05 for all). There were no significant differences between reverters and non-reverters in terms of premorbid IQ, motor severity, LEDD, GDS-15 or anticholinergic use (p>0.05 for all).

PD-MCI subtypes were examined. At baseline, 78% (n=109) were multidomain PD-MCI, while the remaining 22% (n=30) had single domain impairment. The number of multi domains impaired ranged from two to five; 51% were impaired in two domains, 25% in three domains, two participants were impaired in four domains and only one was impaired in all five domains. Executive function was the most commonly impaired domain (59%), followed by memory (51%), attention (35%), language (31%) and visuospatial function (24%). Of those diagnosed with PDD, only 3 had single domain PD-MCI at baseline; 16 had impaired executive function at baseline, 12 had impaired visuospatial function, 11 had impaired memory, and 8 had impaired attention while only 5 had impaired language.

Using PD-MCI level I criteria (figure 1B), a greater proportion of participants were classified as PD-CN compared with level II criteria at baseline, 18 and 36 months (59% vs 34%, χ^2^=26.7, p<0.001; 61% vs 32%, χ^2^=40.1, p<0.001; and 62% vs 35%, χ^2^=35.4, p<0.001, respectively). Between baseline and 18 months, 68% of participants were stable, 12% cognitively declined and 10% improved using PD-MCI level I criteria; 56% of the remaining participants were stable between 18 and 36 months with 16% showing cognitive decline and 7% improvement. Over 36 months, 51% (n=95) were cognitively stable, 13% (n=25) cognitively declined and 8% (n=15) improved.

PD-MCI level I and II criteria classification accuracy was compared using baseline data; PD-MCI 2SD was used to define PD-MCI as it had greater diagnostic stability. In total, 53.2% (n=100) and 16.0% (n=30) were identified as PD-CN and PD-MCI, respectively, using both criteria. Data revealed that 5.9% (n=11) of those defined with level I criteria were false-positive classifications while 25.0% (n=47) were false-negative classifications (χ^2^=22.5, p<0.001). Of those who developed PDD, all participants were identified as PD-MCI using level I criteria and 15 (83%) were identified using level II criteria with a 2SD cut-off.

## Discussion

To the best of our knowledge, this is the first study to investigate the stability of PD-MCI using MDS level I and II criteria over time. Using level II criteria, we found that more than a quarter of participants were cognitively stable over 36 months, one-third cognitively declined and 14% improved. Eight per cent of participants developed PDD, of whom most were previously classified as PD-MCI 2SD. We also found that more than three-quarters had multidomain impairment, and 83% of PDD participants had multidomain impairment at baseline.

Our results show that the operational definition of PD-MCI is an important consideration. Using level I criteria may be more clinically convenient—it is quick to administer and, as our results show, comparably stable. We applied an MoCA score of <26 plus subjective cognitive decline to classify PD-MCI, which gave a reasonable indication of patients who had PD-MCI. We did not use the MMSE to classify cognitive impairment as previous studies have suggested that the psychometric properties of the MMSE are not sensitive to PD-MCI.^15^ ^16^

However, one-quarter of patients were subject to type I error using level I criteria compared with using the more comprehensive level II criteria and stricter cut-offs. We used a schedule of 11 cognitive assessments across five cognitive domains with cut-offs of 1, 1.5 and 2SD, below normative values; using the 2SD cut-off gave greater diagnostic certainty. One study with a smaller sample size (n=76) found that using a 1.5SD PD-MCI cut-off, 13% of participants improved, 3% fluctuated and 22% cognitively declined.^17^ In comparison, our data showed that a greater proportion of participants fluctuated (19%), the majority of whom were classified as PD-MCI using a 1 or 1.5SD cut-off, whereas a 2SD cut-off was more stable. This could suggest that PD-MCI 2SD has greater diagnostic certainty. A previous study suggested that 2SD below normative values had optimal sensitivity and specificity compared with 1 or 1.5SD.^3^

We demonstrated that 7–10% of patients classified as PD-MCI reverted back to normal at 18 and 36 months (18% cumulatively), although this rate was much lower using a more conservative 2SD cut-off. A prospective study found a similar conversion rate of MCI to normal cognition (9%) in participants with persistent PD-MCI at time of diagnosis compared with 3-year follow-up.^18^ Reversions to normal cognition from PD-MCI may be due to a learning effect, an effect of medication or normal fluctuations in cognition.^7^ In a non-PD population, 38% of people with MCI reverted to normal cognition over a median of 5.1 years; fewer participants reverted if they had amnestic MCI or multidomain MCI and poorer cognitive function.^19^ Poorer cognitive functioning in non-reverters is consistent with the findings of our study; we found that fewer participants reverted if they had multidomain PD-MCI and were impaired in executive function. Roberts *et al* 19 reported that reverters were nearly seven times more likely to later develop MCI or dementia than those with baseline normal cognition, suggesting that MCI at any time point may have prognostic utility.

The strengths of this prospective study are its longitudinal design, the use of an incident cohort of community-representative patients with PD, and the comprehensive schedule of neuropsychological tests used. As with any longitudinal study, missing data were an issue. This has implications for classification of PD-MCI and could result in a type II error classification of some participants. However, we examined differences between participants at baseline and those with missing data were found to be representative of the whole sample.^7^ A small number of participants did not return for further assessments; these participants may have been pertinent to the findings of this study as they may have had more rapid decline in terms of cognition and disease progression. However, there were no significant differences in baseline scores. Age and education are factors that may affect cognition. We examined the scores and cut-offs for cognitive tests using age and education as covariates. However, remodelling our data did not have a significant impact on PD-MCI classification.

In conclusion, we have shown that PD-MCI is complex and subject to fluctuation over time, which increases diagnostic uncertainty. PD-MCI level I criteria may have greater clinical utility but more comprehensive level II criteria with 2SD cut-offs provide greater prognostic utility. We propose that clinicians could apply level I criteria using an MoCA score of <26, which would help to identify PD-MCI, and would have some prognostic value in identifying patients at risk of developing PDD. However, clinicians should be cautious when using the MMSE as scores may not be sensitive to cognitive impairment in PD.
